# Apoptosis in the liver of male db/db mice during the development of obesity and type 2 diabetes

**DOI:** 10.18699/VJ20.43-o

**Published:** 2020-07

**Authors:** S.V. Michurina, I.Yu. Ishchenko, S.A. Arkhipov, M.A. Cherepanova, D.V. Vasendin, E.L. Zavjalov

**Affiliations:** Research Institute of Clinical and Experimental Lymphology – Branch of the Institute of Cytology and Genetics of Siberian Branch of the Russian Academy of Sciences, Novosibirsk, Russia; Research Institute of Clinical and Experimental Lymphology – Branch of the Institute of Cytology and Genetics of Siberian Branch of the Russian Academy of Sciences, Novosibirsk, Russia; Research Institute of Clinical and Experimental Lymphology – Branch of the Institute of Cytology and Genetics of Siberian Branch of the Russian Academy of Sciences, Novosibirsk, Russia; Research Institute of Clinical and Experimental Lymphology – Branch of the Institute of Cytology and Genetics of Siberian Branch of the Russian Academy of Sciences, Novosibirsk, Russia; Siberian State University of Geosystems and Technologies, Novosibirsk, Russia; Institute of Cytology and Genetics of Siberian Branch of the Russian Academy of Sciences, Novosibirsk, Russia

**Keywords:** db/db mice, obesity, type 2 diabetes mellitus (DM2), liver, endothelial cells, hepatocytes, Bcl-2, Bad, мыши db/db, ожирение, сахарный диабет 2-го типа, печень, эндотелиоциты, гепатоциты, Bcl-2, Bad

## Abstract

Obesity and diabetes mellitus are known to lead to the development of metabolic syndrome and non-alcoholic fatty liver disease (NAFLD). The mechanisms of programmed cell death are actively involved in maintaining cellular homeostasis along development of NAFLD. Proteins of the BCL-2 family are key regulators of physiological and pathological apoptosis. Homozygous males of BKS.Cg-Dock7mLeprdb/+/+/J mice (db/db mice) are characterized by progressive obesity and the development of type 2 diabetes mellitus (DM2) with severe hyperglycemia at 4–8 weeks and organ lesions at 8–10 weeks of age. The aim of this research was to study the expression of molecular cell regulators of apoptosis in liver cells of db/db mice males at different stages of obesity and diabetes development (at the age of 10 and 18 weeks). Immunohistochemical analysis (using the indirect avidin-biotin peroxidase method) and morphometric evaluation of the expression of the antiapoptotic protein Bcl-2 and the proapoptotic protein Bad in liver cells of studied animals at different stages of obesity and DM2 were carried out. An excess of the value of the Bcl-2 protein staining area over the Bad protein staining area was revealed in the liver of 10-week-old animals. The Bcl-2/Bad expression area ratio in 10-week-old animals was twice as high as in 18-week-old animals, which indicates the presence of conditions for blocking apoptosis in the liver of younger db/ db mice. At the 18th week of life, db/db mice displayed an almost threefold increase in the expression area of the Bad protein against the background of an unchanged expression of the Bcl-2 protein. The decrease in the Bcl-2/Bad staining area ratio in 18-week-old animals was due to the increase in the Bad expression area, which indicates the absence of antiapoptotic cell protection and creates conditions for activation of the mitochondrial pathway of apoptosis in the liver of male db/db mice with pronounced signs of obesity and DM2.

## Abstract (ru)

Известно, что ожирение и сахарный диабет приводят к развитию метаболического синдрома и неалкогольной жировой болезни печени. В поддержании клеточного гомеостаза при неалкогольной жировой болезни печени принимают активное участие механизмы запрограммированной клеточной гибели. Белки семейства BCL-2 являются ключевым регулятором физиологического и патологического апоптоза. Используемые в исследовании гомозиготные самцы мышей линии BKS.Cg-Dock7mLeprdb/+/+/J (мыши db/db) характеризуются прогрессирующим ожирением и развитием сахарного диабета 2-го типа (СД2), выраженной гипергликемией с 4–8-й недели жизни и развитием органных поражений после 8–10-й недели. Целью работы было изучить экспрессию молекулярно-клеточных регуляторов апоптоза в клетках печени самцов мышей db/db на разных сроках развития ожирения и сахарного диабета (в возрасте 10 и 18 нед). Проведены иммуногистохимический анализ (с помощью непрямого авидин-биотинового пероксидазного метода) и морфометрическая оценка экспрессии антиапоптотического белка Bcl-2 и проапоптотического протеина Bad в клетках печени исследуемых животных на разных сроках развития ожирения и СД2. В печени исследуемых самцов в возрасте 10 нед установлено превышение значения площади окрашивания на белок Bcl-2 над белком Bad. Индекс соотношения площадей экспрессии Bcl-2/Bad у 10-недельных животных оказался в два раза выше по сравнению с 18-недельными особями, что свидетельствует о наличии условий для блокирования процессов апоптоза в печени мышей db/db более раннего возраста. На 18-й неделе жизни у самцов мышей db/db обнаружено почти трехкратное увеличение площади экспрессии белка Bad на фоне неизменившейся экспрессии белка Bcl-2. Снижение значения соотношения площадей окрашивания Bcl-2/Bad у 18-недельных животных произошло за счет роста площади экспрессии Bad, что подтверждает отсутствие антиапоптотиче- ской защиты клеток и создает условия для активации митохондриальной «ветви» апоптоза в печени самцов мышей db/db с выраженными признаками ожирения и СД2.

## Introduction

Mechanisms of programmed cell death are actively involved in maintaining cell homeostasis in the development of nonalcoholic fatty liver disease (NAFLD) (Schuppan, Schattenberg, 2013). Obesity and related metabolic disorders, including lipid accumulation in the liver and inflammation, play an important role in liver carcinogenesis. Recent data indicate that obesity and diabetes lead to the development of metabolic syndrome and NAFLD, which can progress in patients with this disease to non-alcoholic steatohepatitis, which includes the risk of cirrhosis and hepatocellular carcinoma (Shimizu et al., 2011). Proteins of the BCL-2 family are key regulators of physiological and pathological apoptosis. According to the modern model of apoptosis regulation, the ratio of the apoptosis regulator proteins Bcl-2, Bad and Bax determines the sensitivity of cells to the effects of apoptotic factors and is a “molecular switch” that determines whether tissue growth or atrophy will occur (Sun et al., 2015). Molecular features of the development of the mitochondrial pathway of apoptosis in the liver of male db/db mice in postnatal ontogenesis at different development stages of obesity and type 2 diabetes mellitus (DM2) have not yet been studied.

The aim of this research – to study the expression of apoptosis molecular cell regulators from the BCL-2 family proteins: the antiapoptotic protein Bcl-2 and the proapoptotic protein Bad in liver cells of male db/db mice at different stages of obesity and DM2 development (at the age of 10 and 18 weeks).

## Materials and methods

The experiments were carried out in the SPF Vivarium of the Institute of Cytology and Genetics, SB RAS, on homozygous males of BKS.Cg-Dock7m+/+Lepr db/J mice (db/db mice). Homozygous individuals of this strain have a defect of the leptin receptor (spontaneous mutation Lepr db) and are characterized by polyphagia, progressive obesity from 3–4 weeks of life, severe hyperglycemia from 4–8 weeks of life, the development of organ lesions after 8–10 weeks. Animals were stored in a room with a regular light cycle (14 h light/10 h darkness), a constant room temperature of 24 ± 2 °C and a relative humidity of 45 ± 10 %. The mice were kept on a standard food (Ssniff, Germany) and water ad libitum.

Studies were conducted on mice aged 10 (n = 7) and 18 (n = 7) weeks, which is comparable to 10 and 18 years of man age, respectively (Flurkey et al., 2006). Animals were sacrificed by cranio-cervical dislocation and liver samples were taken for light-optical and immunehistochemical studies. All experiments were performed in compliance with the principles of humanity and carried out in accordance with the “Rules for the Use of Experimental Animals” (the Annex to the order of the Ministry of Health of the USSR from 12.08.1977, No. 755) and the European Unity Directive (86/609/EEC). The study was approved by the local ethics committee (Protocol No. 128 of 15 March 2017).

The liver pieces were fixed in 10 % buffered formalin (Bio- Vitrum, Russia) for 48 h, dehydrated in a series of alcohols of increasing concentration and enclosed in histomix (BioVitrum). The organ slices 3 μm in thickness were obtained using a LEICA RM2155 microtome (Germany) Liver preparations were stained with Mayer hematoxylin and eosin for light-optical examination.

An immunohistochemical study of the Bcl-2 and Bad protein expression was performed on liver paraffin sections using the indirect avidin-biotin-peroxidase method using the VectaStain Universal Elite ABC Kit (Vector Laboratories, Catalog Number PK-7200). At the last stage, immunohistochemical staining was carried out in a chromogenic substrate containing diaminobenzidine (the solution is prepared ex tempore from the components of the ImmPACT DAB kit, Vector Laboratories, Catalog Number SK-4105). Some sections were stained with Mayer hematoxilin, washed with distilled water and, after dehydration, mounted under the cover glass. To quantify the expression of Bcl-2 and Bad in the mouse liver, a computer-assisted morphometric analysis of digital photographs obtained using a LEICA DM 2500 microscope with a LEICA DFC425C video camera (Germany) at ×400 magnification was performed. Using the Image J software program, the average area of the staining zones on Bcl-2 and Bad was determined on digital images. The ratio of Bcl-2 expression area to Bad expression area was calculated.

Statistical processing of research results was carried out using Statistica 6.1 (serial number AXXR101E832903FA). To analyze the data obeying the normal distribution (the average staining area of Bcl-2 and Bad proteins), the arithmetic mean and standard error of the arithmetic mean were calculated; the significance of differences between the studied groups was established using Student’s t-test. The significance of data differences other than the normal distribution (the ratio of Bcl-2 expression area to Bad expression area) was determined using the nonparametric Mann–Whitney test. Differences between the values compared were considered statistically significant at p < 0.05.

## Results

In the liver of the male mice studied at the age of 10 weeks, stagnations in the interlobular veins, dilatation of lymphatic vessels and bile ducts were detected. Signs of protein dystrophy and lipid accumulations, mainly of small droplet nature, were found in some hepatocytes and in groups of parenchymal cells located mainly in the intermediate zones of the hepatic lobules.

**Fig. 1.**
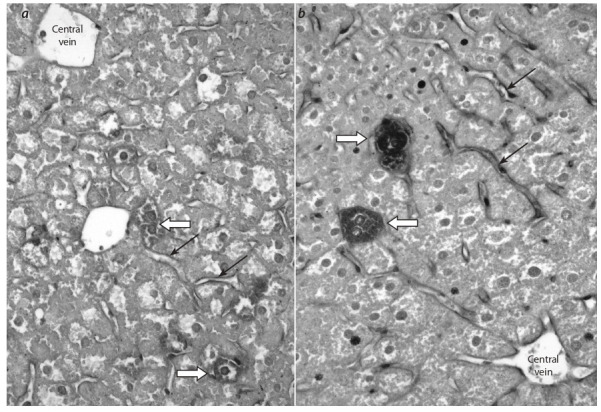
The liver of db/db mice aged 10 weeks: a – poorly marked immunohistochemical staining for the proapoptotic protein Bаd with subsequent Maier’s hematoxylin staining; b – pronounced immunohistochemical staining for the antiapoptotic protein Bcl-2 with subsequent Maier’s hematoxylin staining. Here and in Fig. 3: black arrows point to immunohistochemically colored sinusoidal capillaries in the liver; white arrows point to immunohistochemically colored hepatocytes. Magnification is ×400.

Immunohistochemically, a weak Bаd-positive signal was identified in individual hepatocytes and in the heterogeneous population of sinusoidal cells of liver blood capillaries (Fig. 1, a) involved in the formation of the blood-lymph barrier in the liver, including endotheliocytes, Kupffer cells, Ito cells and Pit cells (Michurina et al., 2016a). At the same time, pronounced immunohistochemical staining was also observed in liver cells for the antiapoptotic protein Bcl-2. In the hepatic lobules, the marker studied was accumulated mainly in the endothelial cells of the lining of blood sinusoidal capillaries and in single hepatocytes (see Fig. 1, b).

**Fig. 2.**
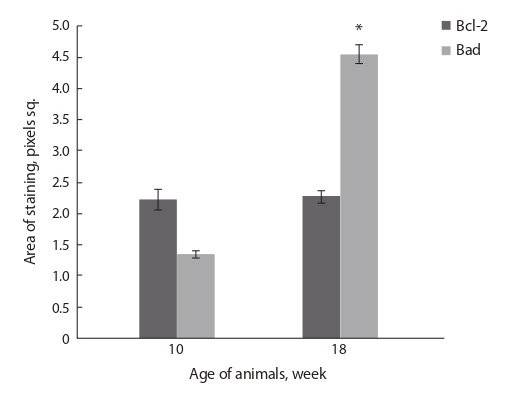
Areas of Bcl-2 and Bad protein staining in the liver of db/db mice aged 10 and 18 weeks. * The differences were significant between groups of “10 weeks” and “18 weeks” ( p < 0.05).

Quantitative evaluation of the expression of the antiapoptotic protein Bcl-2 and the proapoptotic protein Bad showed an excess of the immunohistochemical staining area for the Bcl-2 protein over the value of this parameter for the Bad protein in the liver of db/db mice males aged 10 weeks (Fig. 2).

In the liver of male db/db mice at the age of 18 weeks, signs of nonalcoholic fatty liver disease (NAFLD) development were more pronounced than in animals aged 10 weeks. Diffuse accumulation of medium-sized and large lipid droplets was found in parenchymal cells of all hepatic lobule zones. It developed against the background of disturbances in microcirculation, intraorgan bile transport, a significant dilatation of blood and lymph vessels in the triad system and central veins.

**Fig. 3.**
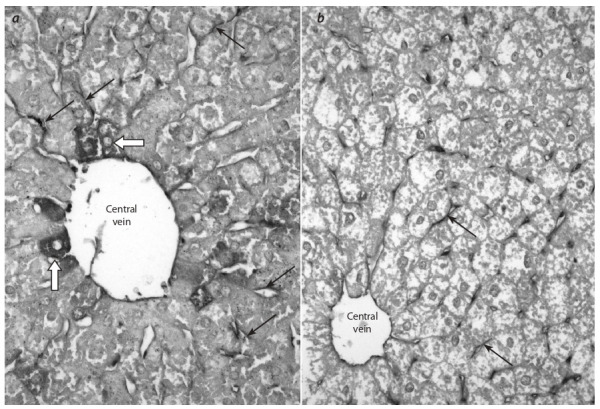
The liver of db/db mice aged 18 weeks: a – pronounced immunohistochemical staining for the proapoptotic protein Bаd with subsequent Maier’s hematoxylin staining; b – weak immunohistochemical staining for the antiapoptotic protein Bcl-2 with subsequent Maier’s hematoxylin staining.

The study of the expression of apoptosis molecular-cell regulators of BCL-2 family proteins in the liver of male db/ db mice at the age of 18 weeks revealed a pronounced immunohistochemical staining for the proapoptotic protein Bad of endothelial cells of blood sinusoidal capillaries. A strong Badpositive signal was detected in hepatocytes located mainly in periportal zones and around central veins (Fig. 3, a) as well as in the ductal epithelium of triad bile ducts. At the same time, weak immunohistochemical staining for the antiapoptotic protein Bcl-2 was detected in cells of the blood-lymph barrier in the liver and in single hepatocytes of еру animals studied at the age of 18 weeks (see Fig. 3, b).

Morphometric analysis of the liver of 18-week-old animals showed an increase in the Bad protein expression area, compared to 10-week-old mice. At the same time, the staining Bcl-2 protein area did not change in comparison with the animals at the age of 10 weeks (see Fig. 2).

**Fig. 4.**
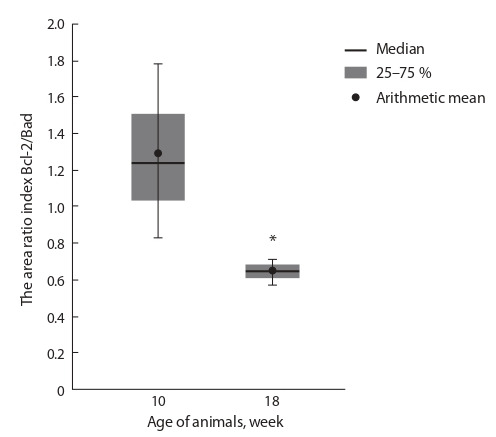
The Bcl-2/Bad staining area ratio. * The differences are significant between the groups “10 weeks” and “18 weeks” ( p < 0.05).

Evaluation of the ratio of Bcl-2/Bad expression areas revealed a significant decrease in this index in 18-week-old db/ db mice compared to 10-week-old animals (Fig. 4), due to an increase, mainly, in the Bad expression area in the animals aged 18 weeks. Data obtained indicate the absence of antiapoptotic cell protection of organ cells, which creates conditions for activation of the mitochondrial pathway of apoptosis in liver cells of the db/db mice at the age of 18 weeks.

## Discussion

It is known that the development of programmed cell death is influenced by posttranslational modifications of BCL-2 family proteins. One of the ways to regulate the activity of apoptosisinducing proteins is the phosphorylation/dephosphorylation process, which affects their ability to form heterodimers with other members of the BCL-2 family proteins. According to current data, the induction of the antiapoptotic protein Bcl-2 expression causes the closure of mitochondrial membrane channels and prevents the release of the protease AIF (apoptosis inducing factor) and cytochrome C, thereby protecting the cell from apoptosis. At the same time, Bcl-2 blocks lipid peroxidation reactions in cell membranes, protecting cells from damage by free radicals and thus preventing the development of apoptosis (Chevalier et al., 2000; Paltsev, 2002; Mushkambarov, Kuznetsov, 2007; Dewanjee et al., 2015). We had previously found that db/db mice were already obese by 10 weeks of age and had severe hyperglycemia with plasma glucose levels of 506 mg/dL (28.1 mmol/L) and higher. There were no significant differences in glucose, triglyceride, total cholesterol, ALT, or GGT levels in the db/db mice aged 10 and 18 weeks (Michurina et al., 2016b). At the same time, as was found in this study, the expression area of the antiapoptotic protein Bcl-2 exceeded the value of the imunohistochemical staining area of the proapoptotic protein Bad in the liver of the 10-week-old animals. Results obtained indicate the presence of antiapoptotic protection of liver cells at this stage of the NAFLD development.

We have previously identified ultrastructural disorders of the energy and protein-synthesis apparatus in liver cells, carbohydrate and fat metabolism disturbances in the liver of 18-week-old male db/db mice with DM2, which leads to the development of protein and fat dystrophy in hepatocytes (Michurina et al., 2016b).

Disorders of blood circulation and lymph flow in the db/ db mouse liver lead to a disruption in the morphological organization of the blood-lymph barrier in the liver, and cause a decrease in LYVE-1 receptor expression on endothelial sinusoid cell membranes. Such morphological rearrangements contribute to the development of tissue hypoxia, oxidative stress and mitochondria damage, which are the inducers of cell death (Eckert et al., 2003; Michurina et al., 2016b). Under these conditions, the mitochondrial pathway of cell apoptosis is launched using the BCL-2 protein family. When the outer membrane of mitochondria is disturbed, a thermolabile factor is also released from the intermembrane space, catalyzing reactions with O2 and leading to the development of oxidative stress. In this case, reactive oxygen species (ROS) are formed that destroy mitochondria and are powerful inducers of apoptosis (Kolesnikov et al., 1999; Dewanjee et al., 2015).

The development of microvesicular steatosis is also considered to be a consequence of severe mitochondrial dysfunction (Begriche et al., 2011). The same mitochondrial disorders are thought to be a common cause of small-bubble steatosis and apoptosis development in obese mice (Trak- Smayra et al., 2011).

In this study, we identified the greatest changes in the endothelial cells of the liver blood sinusoidal capillaries. We are just beginning to understand the complexity of the endothelial cell functions. It is now proven that these cells control liver regeneration as “a spatiotemporal rheostat”. Dynamically regulating the angiopoietin-2 expression, they coordinate their own regeneration and proliferation of hepatocytes, support the restoration of connective tissue, and control the maturation and resting state of blood vessels (Hu et al., 2014). The endothelium acts as the first line of defense against invasion by pathogenic microorganisms, and also regulates vascular tone and permeability. Since damaged endotheliocytes can separate from their basement membrane and circulate freely in the blood, the possibility of detecting endothelial apoptosis in vivo was discussed. The degree of development of vascular injuries directly correlates with organ trauma in critically ill patients (Hutchins et al., 2013). The pronounced immunohistochemical staining revealed by us in the liver of the 18-week-old male db/db mice for the proapoptotic protein Bad of endothelial cells of the blood sinusoid capillaries with a low level of the antiapoptotic protein Bcl-2 expression in them indicates the development of a mitochondrial pathway of apoptosis in the cells of the blood-lymph barrier in the liver under NAFLD (Shimizu et al., 2011; Hutchins et al., 2013).

Since apoptosis is triggered through the inactivation of Bcl-2 upon its binding to the Bad protein, the increase in the proapoptotic Bad staining area established by us indicates the absence of antiapoptotic protection and the apoptosis development along the mitochondrial pathway in liver cells. This is also confirmed by a decrease in the Bcl-2/Bad liver expression area ratio in the male db/db mice at the 18th week of life.

## Conclusion

Immunohistochemical analysis and morphometric evaluation of the expression of apoptosis molecular-cell regulators of BCL-2 family proteins – the antiapoptotic protein Bcl-2 and the proapoptotic protein Bad – in the liver cells of male db/ db mice were carried out at different stages of obesity and type 2 diabetes mellitus development. An excess of the value of the Bcl-2 protein staining area over the Bad protein staining area was revealed in the liver of 10-week-old animals. The Bcl-2/ Bad expression area ratio was twice as high in the 10-week-old animals as in the 18-week-old animals, indicating the presence of conditions for blocking apoptosis in the liver of younger mice. At the 18th week of life, mice displayed an almost threefold increase in the expression area of the Bad protein against the background of an unchanged expression of the Bcl-2 protein. The decrease in the ratio of Bcl-2/Bad staining areas in the 18-week-old animals was due to the increase in the Bad expression area. The obtained results indicate the absence of antiapoptotic cell protection and the creation of conditions for activation of the mitochondrial pathway of apoptosis in the liver of the male db/db mice with pronounced signs of obesity and DM2.

## Conflict of interest

The authors declare no conflict of interest.
